# Single- and Bayesian Multi-Marker Genome-Wide Association for Haematological Parameters in Pigs

**DOI:** 10.1371/journal.pone.0159212

**Published:** 2016-07-19

**Authors:** Siriluck Ponsuksili, Henry Reyer, Nares Trakooljul, Eduard Murani, Klaus Wimmers

**Affiliations:** 1 Research Unit ‘Functional Genome Analyses’, Institute for Genome Biology, Leibniz Institute for Farm Animal Biology (FBN), Wilhelm-Stahl-Allee 2, D-18196, Dummerstorf, Germany; 2 Research Unit ‘Genomics’, Institute for Genome Biology, Leibniz Institute for Farm Animal Biology (FBN), Wilhelm-Stahl-Allee 2, D-18196, Dummerstorf, Germany; China Agricultrual University, CHINA

## Abstract

Haematological traits are important traits that show associations with immune and metabolic status, as well as diseases in humans and animals. Mapping genome regions that affect the blood cell traits can contribute to the identification of genomic features useable as biomarkers for immune, disease and metabolic status. A genome-wide association study (GWAS) was conducted using PorcineSNP60 BeadChips. Single-marker and Bayesian multi-marker approaches were integrated to identify genomic regions and corresponding genes overlapping for both methods. GWAS was performed for haematological traits of 591 German Landrace pig. Heritability estimates for haematological traits were medium to high. In total 252 single SNPs associated with 12 haematological traits were identified (NegLog10 of p-value > 5). The Bayesian multi-marker approach revealed 102 QTL regions across the genome, indicated by 1-Mb windows with contribution to additive genetic variance above 0.5%. The integration of both methods resulted in 24 overlapping QTL regions. This study identified overlapping QTL regions from single- and multi-marker approaches for haematological traits. Identifying candidate genes that affect blood cell traits provides the first step towards the understanding of the molecular basis of haematological phenotypes.

## Background

Genome-wide associational study (GWAS) has become a powerful genomics tool for mapping genetic loci associated with common diseases and quantitative traits. Haematological traits are important in the sense that they can reflect the immune status and healthy conditions and be utilized as biomarkers in human and animals [1]. Pigs are valuable as agricultural commodities and share some similarities in physiology and genome as well as haematological traits with humans. Therefore pigs can serve as a tractable model to study genetic determination of physiological and metabolic traits [2, 3].

Haematological traits include three components, leukocytes, erythrocytes and platelets, which are markers of immune and/or inflammatory responses [4, 5]. A few studies have reported on QTL mapping and GWAS for haematological traits in the pig and most studies used F2 resource populations created by mating two genetically distinct breeds [6–9]. Two studies of purebred pigs are available, one has detected QTL affecting haematological traits based on a linkage analysis using 206 microsatellite markers [10] and the other identified SNPs associated with haematological traits by GWAS [11]. GWAS results for haematological traits at three growth stages in a White Duroc X Erhualian F2 intercross were also reported [12].

In the present study, we report GWAS for haematological traits of 591 performance-tested pigs from commercial herds of German Landrace (DL) using the PorcineSNP60 BeadChip (Illumina Inc., San Diego, CA, USA). The Bayesian multi-marker approach was integrated with single-marker regression analyses to identify genomic regions and corresponding genes overlapping for both methods.

## Materials and Methods

### Animals and sample collection

Animal care and tissue collection procedures followed the guidelines of the German Law of Animal Protection, and the experimental protocol was approved by the Animal Care Committee of the Leibniz Institute for Farm Animal Biology (FBN). Animals (n = 591) of a German Landrace (DL) herdbook herd were kept at the Experimental Farm of the FBN. Animals were fed ad libitum. Samples were collected from the pigs at an average age of 170 days at the experimental slaughter facility of the FBN after electronarcosis followed by exsanguination. Veterinary inspection of the animals before and of carcasses and organs after slaughter ensured that only animals without any impairment, disease symptoms or inflammatory and pathological signs were considered, thus avoiding any bias of blood phenotypes. Haematological traits (White blood cell count, WBC; Lymphocytes count, LYM; Red blood cell count, RBC; Haemoglobin concentration, HGB; Haematocrit level, HCT; Mean Corpuscular Volume, MCV; Mean Corpuscular Haemoglobin, MCH; Mean Corpuscular Haemoglobin Concentration, MCHC; Red Distribution Width, RDW; Platelets, PLT; Mean Platelet Volume, MPV; Plateletcrit, PCT; Table 1) were determined using an automated blood analyser device (ABX Pentra 60 HORIBA, Montpellier, France). Liver tissues were sampled in order to extract DNA using the QIAamp DNA Mini Kit (Qiagen, Hilden, Germany).

**Table 1:** Number of samples, means, standard deviations, variance components and estimates of heritability for haematological traits.

Traits	N	Mean ± SD	σ^2^_e_	σ^2^_a_	h^2^
WBC (10³/mm³)	558	20.8 ± 4.9	17.98	5.33	0.23
LYM (#)	567	7.6 ± 1.9	1.66	1.57	0.49
RBC (10^6^/mm³)	567	8.1 ± 0.8	0.39	0.27	0.41
HGB (g/dl)	559	13.7 ± 1.4	1.21	0.80	0.40
HCT (%)	564	43.2 ± 3.9	9.53	4.92	0.34
MCV (μm³)	567	53.4 ± 3.3	3.29	7.22	0.69
MCH(pg)	561	16.9 ± 1.3	0.56	1.12	0.67
MCHC (g/dl)	561	31.7 ± 1.2	0.45	0.90	0.67
RDW (%)	565	16.5 ± 1.9	1.70	1.59	0.48
PLT (10³/mm³)	545	317.5 ± 82.2	4016.67	2522.7	0.39
MPV (μm³)	564	7.5 ± 0.5	0.19	0.11	0.37
PCT(%)	555	0.2 ± 0.1	0.02	0.004	0.17

WBC, White blood cell count; LYM (#), Lymphocytes count, RBC, Red blood cell count; HGB, Haemoglobin concentration; HCT, Haematocrit level; MCV, Mean Corpuscular Volume; MCH, Mean Corpuscular Haemoglobin; MCHC, Mean Corpuscular Haemoglobin Concentration; RDW, Red Distribution Width; PLT, Platelets; MPV, Mean Platelet Volume; PCT, Plateletcrit;. σ2_e_, residual variance; σ2_a_, additive genetic variance; h^2^, heritability.

### SNP genotypes

Genotyping was performed using the PorcineSNP60 BeadChip (Illumina Inc., San Diego, CA, USA) according to the manufacturer's SNP Infinium HD assay protocol. In brief, 200 ng of DNA were amplified, fragmented, and hybridized to the PorcineSNP60 BeadChip containing 62,163 locus-specific 50-mers that are covalently linked to beads distributed on the microarray surface. Single-base extension of captured oligos allowed the incorporation of labelled nucleotides that were detected by Illumina iScan, and images were subsequently converted to intensity data. The intensity data were normalized and the cluster position, genotype, and quality score were assigned using the GenomeStudio software (Illumina Inc.). Quality control steps were applied including removing samples with call rates < 99%. Markers with low minor-allele frequency (< 5%) were excluded. Markers that strongly deviated from Hardy-Weinberg equilibrium (p < 0.0001) were also filtered out. The average call rate for all samples was 99.8% ± 0.2. All markers on the PorcineSNP60 BeadChip were mapped to the porcine reference genome, Sscrofa 10.2.

### Single SNPs GWAS

Haematological traits were analysed for an association with SNPs using a mixed-model analysis of variance in JMP Genomics (SAS Institute, Cary, NC, USA). Mixed-model analysis tests an association between traits and a single SNPs and simultaneously adjusts for population structure and family relatedness [13]. The genetic similarity matrix between individuals was first computed as identity by descent of each pair for the k-matrix. This genome wide relatedness and the slaughter day were used as random effects. For controlling of population stratification, the correlation-selected principal components analysis was used [14, 15]. Significant correlations at a false discovery rate (FDR) of 5% were considered as covariates. Additionally, genotype and gender were used as fixed effects, age and carcass weight were considered as covariates. Significantly associated SNP markers were reported at a threshold of NegLog10 (p-value) > 5. In order to consider multiple testing issues, a false discovery rate (FDR) was estimated (FDR < 5% corresponding to NegLog10 (p-value) > 6).

### Bayesian GWAS

Prior to the analyses, the genotype matrix was processed using fastPHASE (version 1.2) to impute missing genotype information [16]. Bayesian models implemented in GenSel software (version 4.55R) were applied to the dataset [17]. All analyses were performed using a chain length of 51000 iterations with the first 1000 cycles being discarded as burn-in. An output was created at every 50th iteration. The proportion of SNP that were considered as having no effect in a single iteration was set to π = 0.995. Thus, approximately 240 SNPs were reported in a single iteration of the Markov chain Monte Carlo (MCMC) sampling. Initially, the Bayes C approach was used to estimate additive genetic and residual variance components for each trait. The heritability was calculated as the proportion of the additive genetic variance to the total phenotypic variance. Subsequently, the prior information of variance components was used to run Bayes B models and estimate SNP effects. For haematological traits, fixed effect of gender was considered as class variable in the models. Age and carcass weight were included as covariates. In addition, estimated SNP effects were combined for all markers located in non-overlapping 1-Mb windows and the window contributions to the genetic variance were estimated using the window option implemented in the GenSel software. In total, 2559 1-Mb windows located on autosomes and the X chromosome were included in the analyses. The theoretical proportion of a single window to the genetic variance of a trait was approximately 0.039% (100%/2559) and 1-Mb windows with contributions above 0.5% were reported.

## Results

### Phenotypic and genotypic measurements

591 pigs of commercial German Landrace (DL) were analysed for the following haematological traits: White blood cell count (WBC), Red Blood Cell Count (RBC), Haematocrit level (HCT), Haemoglobin concentration (HGB), Mean Corpuscular Volume (MCV), Mean Corpuscular Haemoglobin (MCH, ratio of HGB to RBC), Mean Corpuscular Haemoglobin Concentration (MCHC, ratio of HGB to HCT), Red Distribution Width (RDW); Platelets (PLT); Mean Platelet Volume (MPV); Plateletcrit (PCT); and Lymphocytes count (LYM). Means of raw data, variance components and heritability estimates generated by the BayesC analyses for the haematological traits are listed in Table 1. Heritability estimates for haematological traits were medium to high (0.17–0.69).

**Fig 1:**
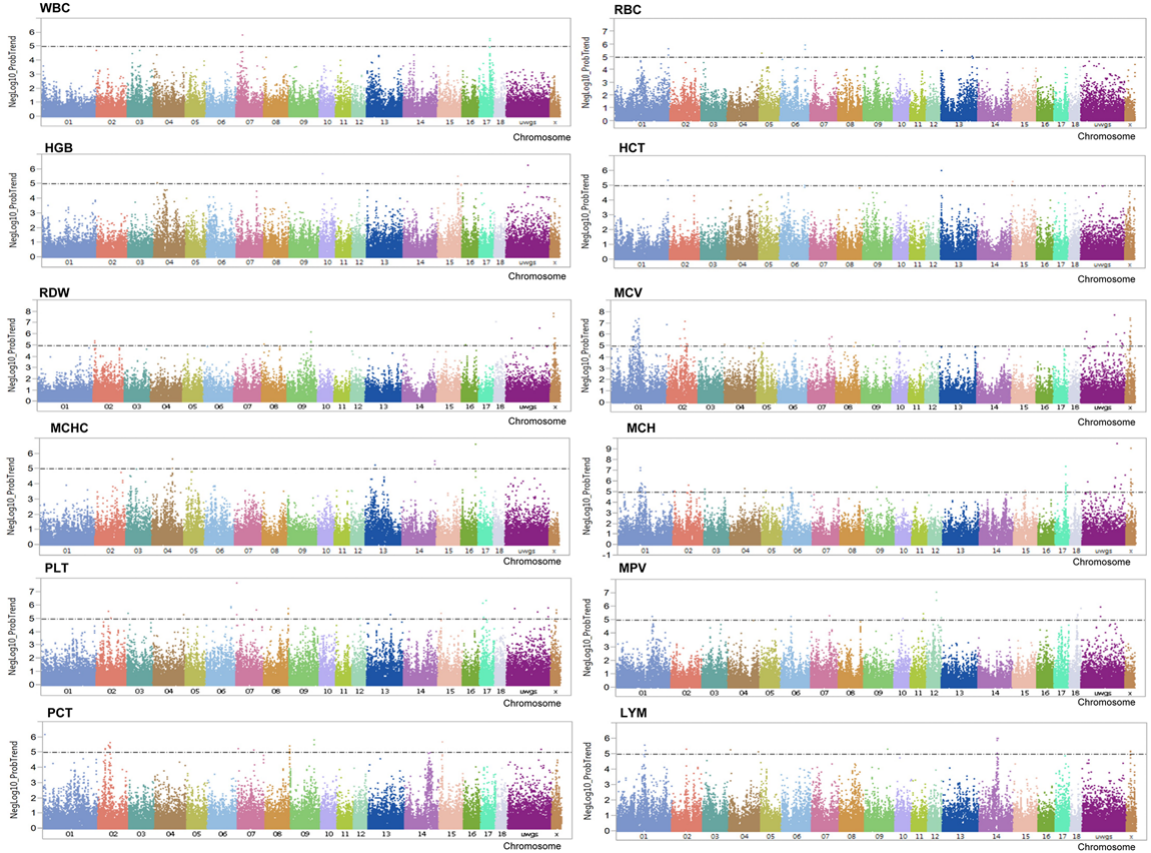
Manhattan plots displaying the genome-wide association based on single-markers analysis with haematological traits in German Landrace. Black lines indicate the significance threshold corresponding to negative log10 (NegLog10)>5.

**Fig 2:**
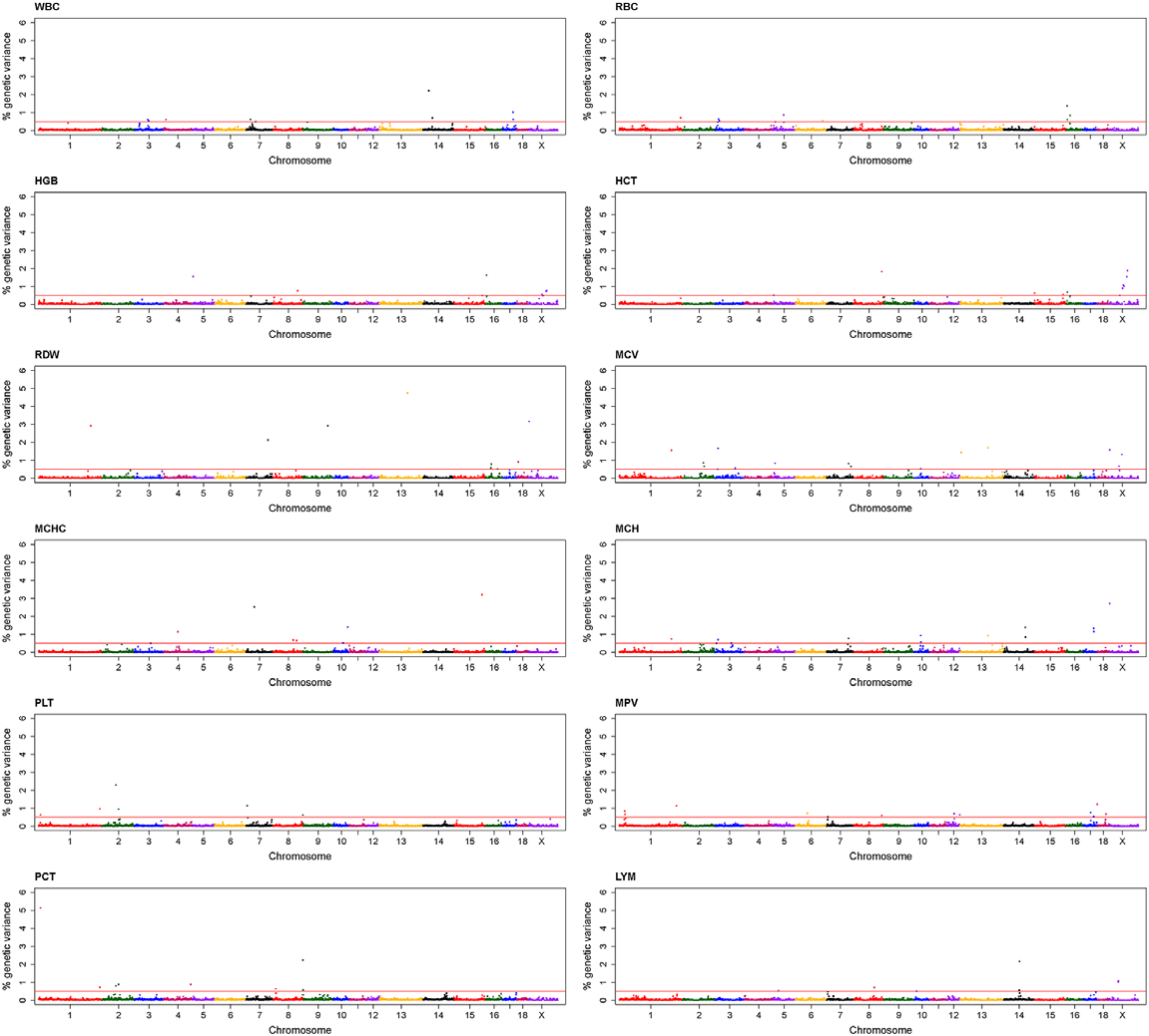
Manhattan plots displaying the genome-wide association based on the Bayesian multi-marker approach (Bayes B) for haematological traits in German Landrace. Horizontal line represents the threshold of 0.05% of additive genetic variance explained by 1-Mb marker windows.

After filtering, 48,909 SNPs were retained for the subsequent GWAS by both single-marker analysis and Bayesian multi-marker approach. For single-marker analysis, a total of 252 SNPs associated with 12 haematological traits were revealed at significance levels of negative log 10 of p value > 5 (See: S1 Table, Fig 1). The top five markers associated with each trait are shown in Table 2 for haematological traits. For the Bayesian multi-marker approach, 1-Mb windows with an explained additive genetic variance of the traits above 0.5% were reported. In total 102 QTL regions were associated with haematological traits that were distributed across the whole genome (See: S2 Table, Fig 2). The regions overlapping between the results of single- (generalized linear model) and multi- (Bayesian approach) marker genome- wide association analyses accounted for 24 out of 102 overlapping QTL regions (See: S2 Table). The transcripts located in the overlapping regions, especially those that showed largest QTL effect for each haematological trait are shown in Table 3.

**Table 2:** Results of single-marker (generalized linear mixed model) genome-wide association analyses in a commercial German Landrace pig population.

Trait	SNP_ID	SSC	position	Candidate genes	Major/minor allele	Variance explained	P-value
HCT	ASGA0055625	13	2696249		A/G	4.20%	9.99E-07
HCT	MARC0069568	1	305211691	NUP214	G/A	3.72%	4.26E-06
HCT	ASGA0068589	15	1138203		A/C	3.64%	5.52E-06
HCT	ASGA0068559	15	666572		G/A	3.48%	8.81E-06
HCT	ALGA0122657	6	136078566		C/A	3.45%	9.63E-06
HGB	ASGA0106305		67212092		A/G	4.43%	5.68E-07
HGB	MARC0018859	10	13257224	LOC100520091	C/A	4.00%	2.10E-06
HGB	ASGA0071068	15	141300399		G/A	3.85%	3.22E-06
HGB	SIRI0000352	15	141355395		A/G	3.85%	3.22E-06
HGB	CASI0009310	4	13581654		A/G	3.53%	8.51E-06
LYM	ALGA0079602	14	78525259		G/A	4.26%	9.49E-07
LYM	ASGA0064978	14	78068318	DDX21	G/A	4.19%	1.01E-06
LYM	ALGA0079529	14	77974347	STOX1	A/G	4.11%	1.30E-06
LYM	ASGA0004304	1	125555348	LIPC	G/A	3.86%	2.75E-06
LYM	H3GA0007030	2	88476158		G/A	3.67%	4.87E-06
MCH	ALGA0004603		153917457		G/A	6.90%	3.39E-10
MCH	ASGA0081192	X	62086511	EFNB1	C/A	6.57%	9.34E-10
MCH	H3GA0049198	17	52392791	SERINC3	A/G	5.30%	4.20E-08
MCH	ASGA0003713	1	95391090		A/G	5.20%	5.81E-08
MCH	MARC0009397	X	51700353	SMC1A	A/G	5.06%	8.83E-08
MCHC	ALGA0116756	16	75218282	GRIA1	G/A	4.72%	2.58E-07
MCHC	ALGA0026794	4	100817218	FCRL3	A/G	3.98%	2.34E-06
MCHC	ALGA0082915	14	146908519	FANK1	G/A	3.89%	3.03E-06
MCHC	M1GA0019565	14	146929488		G/A	3.70%	5.27E-06
MCHC	INRA0040046	13	39012509	CHDH	G/A	3.66%	5.92E-06
MCV	H3GA0055482		143472051		A/G	5.49%	2.03E-08
MCV	ALGA0099736	X	48628584		G/A	5.30%	3.61E-08
MCV	MARC0071761	X	48604940		G/C	5.30%	3.61E-08
MCV	H3GA0002563	1	122922363		A/G	5.25%	4.12E-08
MCV	ASGA0081192	X	62086511	EFNB1	C/A	5.15%	5.62E-08
MPV	ALGA0066401	12	42685899		G/A	5.00%	9.10E-08
MPV	MARC0044698	12	42662420		A/G	4.54%	3.66E-07
MPV	M1GA0024295		45786519		G/A	4.77%	1.09E-06
MPV	H3GA0051240	18	58034747		G/A	4.09%	1.42E-06
MPV	ASGA0051711	11	77040959	LOC100524825	A/G	3.80%	3.45E-06
PCT	M1GA0000623	1	9221985	IGF2R	G/A	4.40%	6.72E-07
PCT	ALGA0054690	9	123960077		A/G	4.13%	1.50E-06
PCT	SIRI0000179	15	15594040		A/G	4.02%	2.12E-06
PCT	ALGA0108179	2	65200938	LOC100515528	A/C	3.97%	2.41E-06
PCT	DIAS0003568	2	65152586	PKN1	G/A	3.97%	2.41E-06
PLT	ASGA0030815	7	5233250	BMP6	A/G	5.66%	2.18E-08
PLT	H3GA0048433	17	34347812		A/G	4.60%	4.82E-07
PLT	H3GA0047920	17	13605158		A/G	4.48%	6.90E-07
PLT	H3GA0053642	6	134996322	LOC102162168	G/A	4.27%	1.27E-06
PLT	H3GA0055786		195877640		G/A	4.19%	1.59E-06
RBC	ALGA0104402	6	136084448		A/C	4.13%	1.14E-06
RBC	M1GA0001919	1	305953354		A/C	3.91%	2.26E-06
RBC	MARC0069568	1	305211691	NUP214	G/A	3.89%	2.39E-06
RBC	ALGA0122657	6	136078566		C/A	3.87%	2.57E-06
RBC	ASGA0055625	13	2696249		A/G	3.81%	3.08E-06
RDW	ALGA0099585	X	38268046		A/G	5.59%	1.48E-08
RDW	ALGA0099588	X	38289148		G/A	5.38%	2.83E-08
RDW	ALGA0096935	18	8952443	ESYT2	G/A	5.00%	8.85E-08
RDW	H3GA0055482		143472051		A/G	4.60%	3.02E-07
RDW	DRGA0009770	9	124492658	TPK1	A/G	4.33%	6.80E-07
WBC	H3GA0020550	7	30832008		A/G	4.10%	1.60E-06
WBC	ALGA0095413	17	52208129	HNF4A	G/A	3.92%	2.74E-06
WBC	ASGA0091117	17	51942244	TOX2	A/G	3.80%	3.98E-06
WBC	ASGA0090286	17	51967501	TOX2	A/G	3.45%	1.14E-05
WBC	MARC0043068	3	65094505		A/G	3.27%	1.91E-05

table shows the top 5 markers for each of the 12 haematological traits

**Table 3:** Results of multi- (Bayesian approach) marker genome-wide association analysis in a commercial German Landrace pig population.

Trait	SSC	start (Mb)	end (Mb)	%Var	#SNPs	Gene located within region and/or overlap with single gene GWAS
HCT	8	139	139.9	1.82	34	FAM13A, HERC3, PYURF
HCT	15	0	1	0.62	27	LOC100739056, NEB
HGB	5	4.1	4.9	1.54	24	PMM1, SCL25A17
HGB	16	5	5.9	1.62	25	MARCH11, FBXL7
LYM	14	77	78	0.55	16	STOX1*
LYM	14	78	79	2.16	28	DDX21*
LYM	X	44.1	45	1.01	14	KDM6A
LYM	X	45	46	1.06	19	miR-221, miR-222
MCH	17	51	52	1.33	29	L3MBTL1*
MCH	17	52	53	1.15	24	TOX2*, MYBL2*, SERINC3*, LOC102159476*
MCH	X	2	2.8	2.72	15	NLGN4X
MCHC	7	40	41	2.53	30	KANK4
MCHC	15	140	140.9	3.21	28	EIF2B1
MCV	1	259	259.9	1.55	15	TLE4
MCV	13	140	140.9	1.7	15	OPA1,TMEM44
MCV	X	2	2.8	1.57	15	NLGN4X
MCV	X	62.1	62.7	1.32	4	EFNB1*
MPV	1	284	285	1.14	27	SNX30
MPV	12	62	63	0.64	30	CENPV*
MPV	18	0	1	1.23	19	
MPV	18	43	43.9	0.67	21	BMPER*, LOC102163798*
PCT	1	9	10	5.13	29	IGF2R*, MAS1,PARK2
PCT	2	69.1	69.8	0.79	8	ZGLP1*, ICAM3, CDC37
PCT	9	0	1	2.23	39	
PLT	2	69.1	69.8	2.3	8	ZGLP1*
PLT	7	5	6	1.14	30	BMP6*, BLOC1S5
RBC	1	305.1	306	0.7	39	NUP214*
RBC	6	136	137	0.53	17	RAVER2*
RBC	16	5	5.9	1.36	25	MARCH11, C7H14orf2
RDW	1	259	259.9	2.92	15	TLE4
RDW	7	108.1	109	2.13	15	LOC100737221
RDW	9	124.1	124.6	2.92	13	TPK1*
RDW	13	140	140.9	4.74	15	OPA1,TMEM44, LOC100627758
RDW	X	2	2.8	3.16	15	NLGN4X
WBC	14	28	29	2.21	29	TMEM132C
WBC	17	51	52	1.01	29	TOX2*

table shows QTL regions that explain the highest proportion of the genetic variance or overlap with single-marker analysis for haematological traits.

*the candidate gene which overlap between single- (generalized linear mixed model) and multi- (Bayesian approach) marker genome- wide association analysis

### White blood cell count and Lymphocytes count

Regarding the analysis of GWAS for WBC, only 3 SNPs located on chromosomes 7 and 17 reached the threshold levels of 5% FDR. The most significant markers associated with WBC located on SSC7 (30.8 Mb; H3GA0020550). The other 2 SNPs (ALGA0095413 and ASGA0091117) located on SSC17 (51.9–52.2 Mb) in the vicinity of the TOX high mobility group box family member 2 (*TOX2)* gene locus (P = 1.6x10^-6^ and P = 2.7x10^-6^) as shown in Manhattan plots of WBC (Fig 1). Bayesian multi-marker approach detected the region that explained 2.2% of the additive genetic variance for WBC at 28–29 Mb on SSC14 (Fig 2). The overlapping region with single-marker analysis was also found on SSC17 (51–52 Mb) and explained 1.01% of the additive genetic variance for WBC. The top 4 LYM associated SNPs mapped on SSC14 (77.9–78.5 Mb) in DEAD-box helicase 21 (*DDX21)* and storkhead box 1 (*STOX1)* and on SSC1 in lipase C, hepatic type (*LIPC)* (Table 2 and Fig 1). It is noteworthy that at the same region on SSC14, QTLs for LYM were identified by Bayesian method, which explained 2.16% of the additive genetic variance (Fig 2). The other two overlapping regions on SSCX were detected by the Bayesian analysis and by the single-marker analysis for an association with LYM. These regions contained the interesting transcripts of lysine demethylase 6A (*KDM6A)* and micro RNA (miR-221 and miR-222) (Table 3).

### Red blood cell traits

We performed GWAS for the following seven erythrocyte-related traits: RBC, HCT, HGB, MCV, MCH, MCHC and RDW. The single-marker approach revealed a number of genetic loci that were significantly associated with RBC (9 loci), HCT (1 loci), HGB (4 loci), MCV (98 loci), MCH (78 loci), MCHC (5 loci) and RDW (27 loci) at significance thresholds of FDR < 5% and NegLog10 (p-value) > 5. The detailed information is shown in additional file 1: S1 Table. In addition, the corresponding Manhattan plots are shown in Fig 1 and the top 5 most significant associations are listed in Table 2.

Interestingly, overlaying the results from both analysis methods consistently discerned RBC associated regions located on SSC1 (305.1–305.2 Mb) physically linked to nucleoporin 214 (*NUP214)*, and on SSC6 (136 Mb) in ribonucleoprotein, PTB binding 2 (*RAVER2)*. The QTL region for RBC with the largest effect was found on SSC16 (5–5.9 Mb) explaining 1.36% of the additive genetic variance (Fig 2). This region was annotated with the transcript of membrane associated ring-CH-type finger 11 (*MARCH11)* and *C7H14orf2*. The SNPs on *NUP214* also associated with HCT (p = 4.2x10^-6^). A region with the largest QTL effect for HCT accounting for 1.82% of the additive genetic variance encompassed the potential candidate genes family with sequence similarity 13 member A (*FAM13A)*, HECT and RLD domain containing E3 ubiquitin protein ligase 3 (*HERC3)* and PIGY upstream reading frame (*PYURF)*. The results also showed some discrepancies between the two GWAS analysis methods such as HGB of which most significant single SNPs were located on SSC15 (141.3 Mb), while multi-marker analysis detected significant loci on SSC5 (4.1–4.9 Mb) and SSC16 (5.0–5.9 Mb).

Most prominent regions revealed from single-marker analyses with MCV and MCH were located on SSCX (46–63 Mb), peaking in the ephrin B1 (*EFNB1)* gene (p = 9.3x10^-10^ for MCH and p = 5.6x10^-8^ for MCV), and on SSC1 (90–105 Mb), covering interesting genes like inhibitor of Bruton tyrosine kinase (*IBTK)* and CD109 molecule (*CD109)*. On SSC17 (50.9–52.9 Mb) the region spanning the l(3)mbt-like 1 (*L3MBTL1)*- MYB proto-oncogene like 2 (*MYBL2)*-*TOX2*- serine incorporator 3 (*SERINC3)* locus showed effect on MCH as revealed by both single- and multi-marker analyses. For MCV a wide QTL region was identified on SSC2 including 4 significantly associated SNP and further supported by two windows exceeding the threshold in Bayesian GWAS (See: S2 Table).

Eleven SNPs significantly associated with MCV, MCH and RDW and located on SSCX (49.5–63.2 Mb), covering the region of *LOC100521307*, Cdc42 guanine nucleotide exchange factor 9 (*ARHGEF9)* and ectodysplasin-A (*EDA)*. By means of multi-marker analysis, a region on SSCX (2–2.8 Mb) explained the most of variance for MCV (1.57%), MCH (2.72%) and RDW (3.16%); the locus was physically linked with the transcript of neuroligin 4, X-linked (*NLGN4X)* (Table 3). Furthermore, we identified single-markers on SSC16 (glutamate ionotropic receptor AMPA type subunit 1(*GRIA1*), SSC4 (Fc receptor like 3 (*FCRL3*) and SSC14 (fibronectin type III and ankyrin repeat domains 1 (*FANK1*) associated with MCHC. In contrast, multi-marker analysis of MCHC revealed QTL on SSC7 (40–41 Mb) and SSC15 (140.0–140.9 Mb) which cover KN motif and ankyrin repeat domains 4 (*KANK4)* and eukaryotic translation initiation factor 2B subunit alpha (*EIF2B1)*.

### Platelet traits

We performed GWAS for three platelet traits: PLT, MPV and PCT. In total, we found 29 PLT loci, 16 MPV loci and 21 PCT loci (See: S1 Table and Fig 1) for single-marker GWAS. The most significant SNPs associated with MPV were MARC0044698 and ALGA0066401 of SSC12 (42.6 Mb) with p = 3.7x10^-7^ and p = 9.1x10^-8^. By multi-marker analysis, QTL with the largest effect identified was on SSC18 at 0–1 Mb, follow by SSC1 at 284–285 Mb which explained 1.23% and 1.14% of the additive genetic variance of MPV, respectively. The SNP ASGA0030815 on bone morphogenetic protein 6 (*BMP6)* (SSC7) and MIGA0002922 on zinc finger, GATA-like protein 1 (*ZGLP1)* (SSC2) were associated with both PLT and PCT. Moreover, the region on SSC2 (69.1–69.8 Mb) was also confirmed by multi-marker analysis (Table 3). It should be noted that two loci significantly associated with PCT hosted candidate genes with biologically plausible functions. The M1GA0000623 SNP within the insulin like growth factor 2 receptor (*IGF2R)* gene (SSC1) was highly associated with PCT with p = 6.7x10^-7^; this region (SSC1, 9–10 Mb) was also confirmed by the multi-marker analysis with the greatest effect that explained 5.13% of the additive genetic variance and hosting 2 interesting transcripts *IGF2R* and MAS1 proto-oncogene (*MAS1)* (Table 3).

## Discussion

The proportion of phenotypic variance explained by markers is considered as a measure of heritability and therefore indicates whether these traits are heritable. Heritability estimates for haematological traits in our study were shown to be moderate for WBC (0.23) which is in line with previous studies in Large White and Yorkshire pigs [18, 19]. Our findings showed that erythrocyte-related traits RBC, HCT, HGB, and RDW were moderately heritable (0.34 to 0.48) compared to MCV, MCH and MCHC which were highly heritable (0.67–0.69). However, in another genetic background RBC and HCT were highly heritable (0.56–0.62) compared to MCV and MCH which were moderately heritable (0.37–0.47), as reported by Mpetile and colleague [19]. The discrepancies of heritability estimation could be due to genetic differences in the breeds studied and the age of the animals when the phenotypic variance was measured.

In total, the single-marker GWAS by GLM analysis revealed 252 SNPs associated with 12 haematological traits at significance levels of 5% FDR, whereas the Bayesian approach detected altogether 102 QTL regions across the genome for 12 haematological traits using a 1- Mb window and the genetic variance above 0.5%. The multi-markers method was challenged to avoid false positives and overestimation of QTL effects derived from single-SNP analysis; accordingly, Bayesian approaches involving SNP-windows to reflect QTL have been applied in many studies before [20–22]. To take advantage of the Bayesian approach and to enhance the power of finding potential candidate genes, we combined the results from both methods by overlaying the derived QTL.The integration of both methods resulted in 24 overlapping QTL regions. For lymphocyte count, two overlapping regions from both methods located on SSCX (44.1–46.0 Mb) at *KDM6A*, miR-221 and miR-222, and SSC14 (77–79 Mb) at *STOX1* and *DDX21*. *KDM6A* (histone 3 lysine 27 demethylase UTX) was identified as a novel regulator for haematopoietic cell migration by using haematopoietic stem and progenitor cell [23]. Consistency of results between both methods was also found for WBC on SSC17 (51–52 Mb) at *TOX2*. *TOX2* is a transcription factor belonging to the TOX family that shares a highly conserved high mobility group DNA-binding domain with the other TOX members. As recently reported, *TOX2* regulates human natural killer cell development by controlling T-BET expression [24]. Recently, a study demonstrated that transcriptional regulator TOX is required for the in vivo differentiation of common lymphoid progenitors into innate lymphoid cells [25]. All together there is growing evidence promoting *TOX2* as a candidate gene for WBC counts.

QTL for MCH and MCV were reported in many pig chromosomes in particular on SSC2 at 55–60 Mb [11] and SSC8 at 42–73 Mb which covers the KIT gene [9, 10, 26]. KIT regulatory mutations are responsible for the dominant white phenotype in pigs [27] and have profound pleiotropic effects on peripheral blood cell measures in Western commercial crossbred pigs and experimental crosses [6, 28, 29]. Inconsistently, others have not found any significant association between the KIT mutations and haematological parameters [30, 31]. Also, in our study, no significant associations with red blood cell traits were detected for both of these regions, in particular not of the KIT region. Landrace pigs have solid white coat color phenotypes and are usually homozygous for the dominant white allele (I) at the KIT gene. Thus there is not QTL segregating; consequently there are no significant SNPs found around the KIT region.

In this study, a region on SSC8 at 128 Mb was found significantly associated with MCV by means of single-marker analysis. Interestingly, Bayesian approaches persistently detected an associated effect for MCV, MCH, and RDW on SSC1, SSC13 and SSCX which constitute some plausible candidate genes contributing to red blood cell traits via haematopoietic mechanisms including *EFNB1*, *TLE4* and *OPA1*. In particular, identified regions on SSCX at 2.0–2.8 Mb and 62.1–62.7 Mb include *NLGN4X* and *EFNB1* as interesting candidate genes. *NLGN4X* encodes a protein which belongs to a family of neuronal cell surface and causal factors for monogenic autism as well as directly impacts neurodevelopmental processes during the formation of neurons and their connections [32, 33]. *EFNB1* encodes a membrane protein that acts as a ligand for Ephrin receptor tyrosine kinases and thus play a potential role in modulating blood pressure [34]. *EFNB1* was detected in leukaemia cell lines and bone marrow and was shown to be involved in normal haematopoietic development and tumorigenesis [35, 36]. The other candidate gene located on SSC1 which involved in the complex processes of haematopoiesis was *TLE4*. The TLE family of genes is a group of highly conserved transcriptional corepressors that are involved in myeloid cell proliferation and survival [37]. Tle4 knockout mice exhibit leukocytopenia, B cell lymphopenia, and significant reductions in haematopoietic stem and progenitor cells [38]. Another plausible candidate locus contributing to red blood cell traits via energy metabolism of erythropoiesis was OPA1 located on SSC13 (140–140.9 Mb).This locus explains 1.7 and 4.7% of the additive genetic variance for MCV and RDW. Erythropoiesis is highly dependent of mitochondrial metabolism through multiple ways [39]. The *OPA1* gene encodes a dynamin-like mitochondrial GTPase OPA1and plays a significant role in mitochondrial structure, maintenance and fusion. In addition, OPA1 –is involved in the regulation of energetic metabolism and cell death, which underscores its multiple physiological roles [40]. Recently, it was reported that when erythropoietic cells are copper deprived, *MFN2* and *OPA1* become up-regulated and functional to promote fusion [41].

In a previous study, the QTLs for RBC were mapped on the end of SSC1 [9]. Regarding the present study, one of common candidate genes in SSC1 (305.1–306 Mb) that associated with HCT and RBC, was *NUP214*. The gene is a member of the FG-repeat-containing nucleoporins and known to be fused with the DEK gene on chromosome 6 in a t(6,9)-translocation associated with acute myeloid leukemia and myelodysplastic syndrome [42].

The highly significant SNPs and regions, that both approaches detected s to be associated with platelet traits (PCT), were physically linked to the *IGF2R* gene (SSC1, 9–10 Mb). The IGFs have an important role in physiologic and neoplastic processes as well as normal and malignant development of the haematopoietic system [43, 44]. Recently, it was reported that deletion of the insulin receptor in murine resulted in an increases platelets count and volume, and blocked the action of insulin on platelet signalling and function [45]. *ZGLP1* (zinc finger, GATA-like protein 1) is a strong positional candidate gene for both PCT and PLT but there is still limited knowledge about the function of this gene. GWAS of PLT further revealed the *BMP6* gene which is the key endogenous regulator of hepcidin, an iron homeostasis gene [46]. In this context, it was also reported that iron deficiency increases megakaryopoietic differentiation and alters platelet phenotype [47].

## Conclusions

In summary, our study provides insights into the genetic architecture of haematological traits and opens new opportunities to the application of haematological parameters as a monitoring indicator for health and infection in pigs. Taking the advantages of Bayesian GWAS approach, combined with a GLM single-marker approach, we were able to provide a list of promising QTL regions and plausible candidate genes that carry common genetic variants associated with RBC, WBC, and platelet phenotypes. Further validation and identification of the causal mutations are necessary. This study provides an additional step towards the understanding of the molecular basis of blood cell phenotypes and could be used as a model for many diseases which might be simpler for blood cell traits rather than most other complex phenotype.

## Supporting Information

S1 Table Results of single-marker genome-wide association analyses for 12 haematological traits. List of significantly associated SNP markers at NegLog10 (p-value) > 5 obtained by single-marker analysis. Haematological traits, marker name, chromosome, position according to Pig Genome Annotation 10.2, major/minor allele, % variance explained, p-value (NegLog10), positional candidate gene.
(XLSX)

S2 Table Results of multi-marker genome-wide association analyses for 12 haematological traits. List of 1-Mb windows with % variance explained > 0.5%. Haematological traits, chromosome, position of start and end of window according to Pig Genome Annotation 10.2, % of the additive genetic variation explained by the QTL regions associated with the traits, number of SNPs in the QTL regions, regions overlapping between the results of single- (generalized linear model) and multi- (Bayesian approach) marker genome- wide association analyses.
(XLSX)
